# Effect of oligonucleotide MT01 delivered by N-isopropylacrylamide modified polyethyleneimine for bone regeneration

**DOI:** 10.3389/fbioe.2023.1204571

**Published:** 2023-06-19

**Authors:** Qian Zhang, Xingyuan Qu, Chen Liang, Hongyan Li, Siyu Du, Chang Wang, Yuandong Xie, Yi Zheng, Lei Wang

**Affiliations:** ^1^ Department of Periodontics, Hospital of Stomatology, Jilin University, Changchun, China; ^2^ Jilin Provincial Key Laboratory of Tooth Development and Bone Remodeling, Jilin University, Changchun, China

**Keywords:** PEN, MT01, bone regeneration, gene therapy, oligodeoxynucleotide

## Abstract

**Objective:** This study aimed to investigate the regulatory effect of N-isopropylacrylamide-modified polyethyleneimine (PEN)-delivered oligodeoxynucleotide (ODN) MT01 on bone regeneration *in vitro* and *in vivo*.

**Methods:** A polyethylenimine (PEI) derivative, PEN, was constructed through Michael addition and employed as a carrier for ODN MT01 transfection. PEN/MT01 nanocomposites were characterized using agarose gel retardation assay, size distribution, zeta potential and transmission electron microscopy. The Cell Counting Kit-8 (CCK-8) assay was used to detect the effect of PEN on cell viability. Alkaline phosphatase (ALP) staining was used to detect the osteogenic differentiation ability of PEN/MT01 nanocomposite. Real-time quantitative PCR (q RT-PCR) and enzyme-linked immunosorbent assay (ELISA) were used to detect the regulatory effects of PEN/MT01 nanocomposite on osteogenic differentiation gene expression. Rat model was observed using the skull defect method and verified using micro-computed tomography (CT), serum biochemical indices, hematoxylin and eosin (H&E) staining and Immunohistochemistry (IHC).

**Results:** PEN had good biological properties and could deliver MT01 well to achieve efficient transmission of MT01. PEN/MT01 nanocomposites were effectively transfected into MC3T3-E1 cells at a ratio of 6.0. CCK-8 assay displayed that PEN had no cytotoxicity to MC3T3-E1 cells. Additionally, PEN/MT01 nanocomposites could promote the expression of osteogenic genes. *In vivo* results revealed that PEN/MT01 nanocomposites could promote bone regeneration more effectively than the other groups.

**Conclusion:** PEN has good biocompatibility and low toxicity, which is a good carrier for ODN MT01. PEN-delivered MT01 can be potentially employed as a useful approach to achieving bone regeneration.

## Graphical Abstract

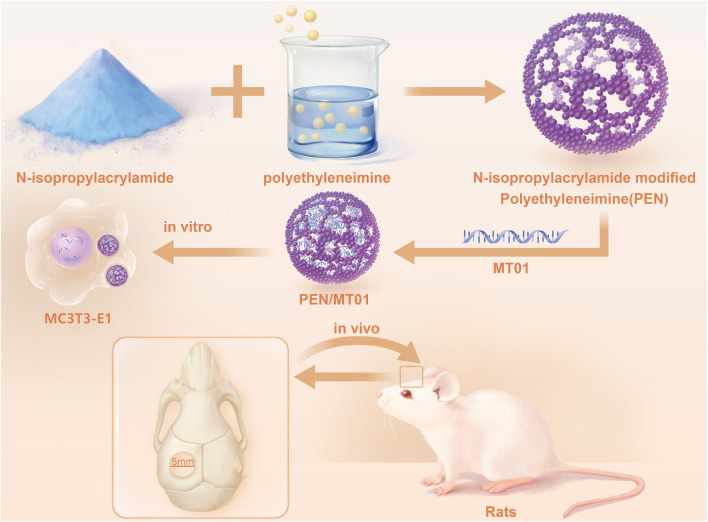

## 1 Introduction

Bone defects remain a challenging common clinical problem in various settings because the potential regeneration of human bone is limited. Bone defects resulting from trauma, chronic infection, congenital defects, surgical resection, or periodontal disease require clinical intervention and autologous bone grafting techniques. However, autologous tissue is scarce and must be harvested from a donor site, which can result in morbidities, including pain, infection, and reoperation (Younger and Chapman, 1989; Elke et al., 2002; John et al., 2003). Recently, with the continuous development of gene therapy, there has been considerable progress in repairing maxillofacial bone defects. Realizing that gene therapy can also treat non-monogenic diseases has expanded its potential. Gene-enhanced tissue engineering is a promising approach for vascularization and osteogenesis (Vega et al., 2021).

Oligodeoxynucleotides (ODN), unmethylated nucleotide core motif sequences, play a significant role in gene therapy (Deng et al., 2011). Compared to traditional small-molecule drugs, ODN depict unique advantages, including realizing personalized drugs and negligible toxicity to normal tissues (Smith and Zain, 2019). MT01 was designed based on human mitochondrial DNA sequences, which is an inhibitory ODN that promotes osteocyte differentiation. Our previous research proved that MT01 could promote osteoblast maturation and activation in rats, reduce rat alveolar bone absorption caused by periodontitis, regulate the expression levels of osteogenesis-related factors such as intracellular runt-related transcription factor 2 (Runx-2), collagen type I (COL I), and osteoprotegerin (OPG), and promote bone marrow mesenchymal stem cell differentiating into osteoblasts. The above research suggests MT01 may regulate bone remodeling (Feng et al., 2011; Wang et al., 2012; Zheng et al., 2017).

Because free ODNs are easily degraded by nucleases (deoxyribonuclease [DNase]) in serum, ODNs with chemical modifications have been studied for clinical application. However, chemical modifications have raised concerns regarding their undesirable side effects. For example, repeated administration of backbone-modified ODNs has been shown to lead to reduced immune responses, lymphoid follicle destruction, and organ enlargement (Nobutaka, 2012). Recently, developing nanotechnology has provided a new strategy to solve the problem of ODN transmission. N-isopropylacrylamide-modified polyethyleneimine (PEN), a derivative of polyethyleneimine (PEI), has the advantages of low toxicity and high loading efficiency (Tian et al., 2012). Xing demonstrated that PEN could protect miR-29a against the nuclease degradation and suppress the migration and invasion of cancer cells measured by wound healing and transwell migration assays (Xing et al., 2020). These findings suggest that PEN could be used as a potential carrier to enable effective gene delivery for the treatment of many diseases.

In the present research, a PEI derivative (namely, PEN) has been constructed through modification of PEI with N-isopropylacrylamide via Michael addition. The derivative PEN was employed as a carrier to achieve MT01 delivery using the MC3T3-E1 cells and rat cranial defects as models. Then, we investigated its ability to induce osteogenesis *in vitro* and *in vivo*.

## 2 Materials and methods

### 2.1 Materials

The mouse osteoblastic cell line MC3T3-E1 cells were obtained from Zhongqiaoxinzhou (Shanghai, China). MT01 and MT01-s (MT01 modified by sulfur) were synthesized by TaKaRa (Dalian, China). The sequence of MT01 is as follows: 5′-ACC CCC TCT ACC CCC TCT ACC CCC TCT-3’. Branched PEI-25K was purchased from Aldrich and used as received. All other chemicals were of the highest reagent grade commercially available and used as received.

### 2.2 Preparation of PEN and PEN/MT01 nanocomposites

PEN derivative was synthesized according to a previously published method (Tian et al., 2012). The PEN solution (adjusted to pH 7.0) and the MT01 solutions were freshly diluted in phosphate buffered saline (PBS) (pH 7.4) to obtain the desired concentrations. Nanocomposites were prepared by gently mixing the carrier PEN and MT01 together at different mass ratios (0.1, 0.2, 0.5, 1.0, 2.0, 4.0, 6.0, 8.0, w/w). Nanocomposites were subsequently incubated at room temperature for 30 min. The final concentration of MT01 was 1 μg/mL.

### 2.3 Cell culture and transfection

MC3T3-E1 cells were propagated to confluence in DMEM (HyClone, United States) containing 10% fetal bovine serum (FBS) (BI, Israel) and 1% penicillin-streptomycin (HyClone, United States), and were maintained at 37°C in a humidified atmosphere of 5% CO_2_. MC3T3-E1 cells were cultured for 24 h; the medium was discarded, and cells were washed twice with PBS (HyClone, United States), MT01 (10 μg/mL), MT01-s (10 μg/mL), PEN/MT01 nanocomposites, and PBS (blank control) were added, and the cells were incubated for 4 h. DMEM (excluding FBS) was added to 2 mL (the final mass concentration of MT01/MT01-s in each well was 1 μg/mL). After 4 h, the medium was discarded, DMEM (containing 10% FBS), and the cells were incubated.

To induce differentiation, cells were seeded in a well. The next day, the original medium was discarded, and the culture medium was replaced with osteogenic induction medium containing 50 mg/mL L-ascorbic acid (Sigma-Aldrich, United States) and 1 M β-glycerophosphate sodium (Solarbio, China), and FBS concentration was 5%. Free-MT01, MT01-s, and PEN/MT01 were added to the medium without FBS for 4 h. The medium was discarded, the cells were washed, and an osteogenic induction medium was added. The osteogenic induction medium was changed with repeated dosing every 2–3 days. Cells were incubated continuously for 7–21 days.

### 2.4 Gel retardation assay

PEN/MT01 nanocomposites were prepared by gently mixing the carrier PEN and MT01 in double-distilled water at different mass ratios (0.1, 0.2, 0.5, 1.0, 2.0, 4.0, w/w) and incubating at room temperature for 30 min before use. The binding ability of PEN to MT01 was then assessed by 2% agarose gel electrophoresis in Tris-acetate-EDTA (TAE) buffer solution (Aladdin, China) (120 V, 22 min). Afterward, the images were photographed and recorded using a gel imager (BIO RAD, United States).

### 2.5 Size distribution and zeta potential

The mean particle sizes and zeta potentials of PEN/MT01 nanocomposites (2.0, 4.0, 6.0, 8.0, w/w) above-mentioned prepared in double distilled water were determined using a Malvern Nano ZS90 Zetasizer (Malvern, United Kingdom). Particle size was assessed from three cycles, and zeta potential was measured by repeated cycles with 100 runs each. The images of PEN/MT01 nanocomposites were measured by transmission electron microscopy (TEM) performing on a 200 KeV Talos F200s microscope (Bruker, Germany).

### 2.6 Cell viability assay

MC3T3-E1 cells (5×10^3^) were cultured in 96-well plates. PEN/MT01 nanocomposites solution with different solute mass ratios (1.0, 2.0, 4.0, 6.0, 8.0, 10.0, w/w) were incubated at 37 °C for 4 h. The control group was treated with PBS. Solution was discarded, DMEM (containing 10% FBS) was added, and the cells were incubated for 24 h. The above procedure was repeated the next day. Cytotoxicity of PEN/MT01 nanocomposites was analyzed using a CCK-8 kit (Invitrogen, United States), and absorbance was recorded at 450 nm using a microplate reader (Bio Tek, United States).

### 2.7 q RT-PCR

A total (2×10^5^) MC3T3-E1 cells were seeded in 6-well plates. Free-MT01, MT01-s, and PEN/MT01 nanocomposites (6.0, w/w) were incubated at 37 °C for 4 h without FBS. The control group was treated with PBS. The medium was discarded, the cells were washed, and an osteogenic induction medium was added. The osteogenic induction medium was changed with repeated dosing every 2–3 days. Cells were incubated continuously for 7 days. Supernatant of 6-well plates was absorbed, and flushed with PBS, 1 mL TRIeasy Total RNA Extraction reagent (Yeasen, China) was added to each well to extract the total RNA in cells of each group, and mass concentration and purity of total RNA samples were tested using NanoDrop2000 spectrophotometer (Thermo Scientific, United States). The cDNA was synthesized using the Hifair^®^ III first strand cDNA Synthesis SuperMix for qPCR (Yeasen, China). ALP, COL I, OCN, and Runx-2 mRNA expression levels in MC3T3-E1 cells cultured for 7 days were amplified using CFX Connect RT PCR Detection System (Bio-Rad, Singapore) with Hifair^®^ qPCR SYBR Green Master Mix (Yeasen, China). Gene expression data were normalized to β-actin and calculated using the 2^^−ΔΔCt^ method. All primers were designed and synthesized by China National Bioengineering Corporation (Shanghai, China). The primer sequences used for amplification are listed in Table 1.

**TABLE 1:** Primer sequence for qRT PCR.

Gene	Sequence of primers (5′→3′)
β-actin	F: CAT​CCG​TAA​AGA​CCT​CTA​TGC​CAA​C
	R: ATG​GAG​CCA​CCG​ATC​CAC​A
ALP	F: GCA​GTA​TGA​ATT​GAA​TCG​GAA​CAC
	R: ATG​GCC​TGG​TCC​ATC​TCC​AC
COL Ⅰ	F: GAC​ATG​TTC​AGC​TTT​GTG​GAC​CTC
	R: GGG​ACC​CTT​AGG​CCA​TTG​TGT​A
OCN	F: AGC​AGC​TTG​GCC​CAG​ACC​TA
	R: TAG​CGC​CGG​AGT​CTG​TTC​ACT​AC
Runx-2	F: TGC​AAG​CAG​TAT​TTA​CAA​CAG​AGG
	R: GGC​TCA​CGT​CGC​TCA​TCT​T

### 2.8 ELISA

The cell culture step is the same as q RT-PCR. Cell culture supernatants were collected and centrifuged, and the cell supernatant biomarkers of bone formation, including ALP, COL I, OCN, and Runx-2 levels, were detected using an ELISA kit from Shanghai Enzyme-linked Biotechnology Company (Shanghai, China), according to the manufacturer’s protocols.

### 2.9 ALP staining and activity

Following a 7-day osteogenic differentiation period, the cells were washed twice with PBS and fixed with 4% paraformaldehyde at room temperature for 15 min. The cells were stained with BCIP/NBT ALP color development Kit (Beyotime, China) at 37 °C for 1 day. All images were captured using a scanner (Epson, United States). To confirm osteogenic differentiation, ALP activity was evaluated, and lysates of RIPA buffer (Beyotime, China) and PMSF (Beyotime, China) at a ratio of 100.0 were collected with a cell scraper, centrifuged at 14,000 rpm, and the supernatant was collected. An ALP assay kit (Beyotime, China) combined with the Enhanced BCA Protein Assay Kit (Beyotime, China) was used to determine ALP activity of each group of cells.

### 2.10 Animal experiment

All experiments were reviewed and approved by the Ethics Committee for Laboratory Animals of the Basic Medical College, Jilin University (ethical approval number: 20210256). The breeding conditions were controlled in strict accordance with GB14925. The light time was 8:00–20:00, the temperature was maintained at 23°C ± 1°C, and humidity was controlled at 50%–60%. The animals were guaranteed sufficient food and drinking water and could eat freely. The feed was maintained for rats. The main ingredients included corn, soybean meal, fish meal, flour, bran, salt, calcium chloride phosphate, stone powder, multivitamins, various trace elements, and amino acids.

In total, 32 with 7-week-old SD male rats were used for the animal experiments. The rats were separated into four groups. Hai’ao^®^ oral cavity repair film (Zhenghai, China) was cut at an average thickness of 0.7–1.0 mm into a circle with a diameter of 5 mm and was sterilized with ultraviolet radiation (254 nm, 70 μW/cm2) for 30 min for standby (Nobutaka, 2012). PEN was set at a concentration of 2 mg/mL, mixed with MT01 (1 mg/mL) at a mass ratio of 6.0, and allowed to stand for 30 min at a 37 °C water bath. Next, 10 μL of PBS, MT01, MT01-s, and PEN/MT01 compounds were added to the tissue surface of the biofilm and freeze-dried. Rats in each group were anesthetized by inhalation of 14% Isoflurane. The skin at the top of the skull was prepared and sterilized, and a full layer incision was made approximately 1.5 cm along the middle suture of the skull top. The skin surface and subcutaneous tissue of the skull were peeled off, and a 5 mm hole was drilled using a hand-held rotary drill (DENTECH, Japan) with copious saline irrigation on the left side of the top skull suture. Avoiding damaging the dura mater. Any remaining debris or bone chips were irrigated gently with sterile saline solution. The biofilm of each group was used to cover the defect. The tissue surface of the biofilm was close to the dura mater. Finally, the incision was sewed closed. Penicillin (160,000 U/day/animal) and Ibuprofen (5 mg/kg/day/animal) were injected intramuscularly for 3 days after the operation.

### 2.11 Serum biochemical test

After 10 weeks of feeding, blood was collected from the orbit of the rats, the levels of glutamic pyruvic transaminase (GPT), glutamic oxaloacetic transaminase (GOT), and urea nitrogen (BUN) were measured using kits (Jiancheng, China).

### 2.12 Micro-CT

Under anesthesia, rats were killed by cardiac perfusion with normal saline. Bone block in skull region of rats was extracted, 10% formaldehyde was added to fix it for 24 h, and then the corresponding three-dimensional image was reconstructed by micro-computed tomography (CT) system (SCANCO µCT, Bruker, Germany). Scanning parameters were 70 kVp, 200 μA, 14 W. Image pro (Media Cybernetics, United States) was used to quantitatively analyze area of new bone and evaluate formation of new bone. The trabecular metric parameters measured included trabecular bone mass density (BMD) and ratio of new bone mass to the detected areas tissue mass (BV/TV).

### 2.13 Histology

Skull samples were taken from rats that died in 10th week and placed in 10% EDTA. Afte days of decalcification, the samples were washed with flowing water, dehydrated with gradient alcohol, embedded in paraffin, and sliced to a thickness of 5 μm. The samples were stained with Hematoxylin and eosin (H&E) and observed under a microscope.

### 2.14 Immunohistochemistry (IHC)

After paraffin sections were dewaxed and hydrated, they were washed three times with PBS (pH 7.4) for 3 min each time. The deparaffinized and hydrated sections were heated in citrate buffer at 121°C for 30 min to retrieve antigenic activity. A 50 μL peroxidase blocking solution was added per slice, and slices were incubated at room temperature for 10 min to block endogenous peroxidase. Then, slices were washed with PBS. A 50 μL of normal nonimmune animal serum was added per slice and incubated at room temperature for 10 min. After removing serum, 50 μL of primary antibody (1:1000, ab23981; Abcam, United Kingdom) was added and incubated at 4 °C overnight, and then rinsed with PBS. A 50 μL of horse radish peroxidase (HRP)-conjugated secondary antibody was added, incubated for 10 min, and then rinsed with PBS. After adding another 50 μL streptomycin avidin peroxidase solution, samples were incubated for 10 min and washed with PBS and DAB solution (100 μL) (MXB, China). Subsequently, samples were rinsed with tap water, counterstained with hematoxylin, and turned blue after rinsing. Afterward, gradient alcohol was used for dehydrating samples, and xylene transparent was applied, sealed with neutral gum, and observed under a microscope (BX51, Olympus, Japan). In the expression analysis of immunohistochemically stained sections of different groups, the same area and the same conditions were selected for gray density analysis using ImageJ software (ImageJ 1.53c, Wayne Rasband, United States).

### 2.15 Statistical analysis

Data analyses were performed using SPSS software (version 18.0); GraphPad v 9.0 software (La Jolla, CA, United States). All data were tested for normality and expressed as mean ± standard deviation (SD). A single factor or multifactor ANOVA Tukey’s method was used to compare the multi-sample measurement data. *p*-values < 0.05 were considered statistically significant.

## 3 Results and discussion

Treating bone defects is a major challenge. ODN has become an effective and widely used method for developing gene therapy (Bhagat et al., 2005; Krieg, 2008; Hidekazu et al., 2015). Our preliminary studies depicted that MT01 (Yang et al., 2010), a synthetic single-stranded ODN, significantly impacted osteogenic proliferation and activation. MT01 strongly inhibits bone absorption and promotes bone formation (Feng et al., 2011; Wang et al., 2012; Zheng et al., 2020). However, owing to the biodegradability of ODN and side effects of chemical modification, a suitable vector is needed to assist transfection and endocytosis of ODN.

### 3.1 Construction and characterization of PEN/MT01 nanocomposites

**FIGURE 1:**
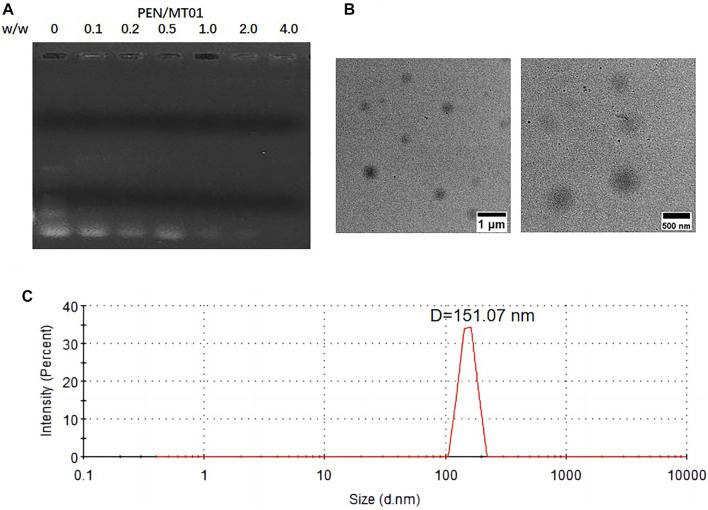
Characterizations of PEN/MT01 nanocomposites. **(A)** Agarose gel retardation assay of PEN/MT01 nanocomposites at various w/w ratios of 0,0.1,0.2, 0.5, 1.0, 2.0, 4.0. **(B)** TEM images of PEN/MT01 nanocomposites with w/w ratio of 6.0. Scale bar:1 μm and 500 nm. **(C)** Size distribution for PEN/MT01 nanocomposites with w/w ratio of 6.0.

The PEN derivative was synthesized by modifying PEI-25K with N-isopropylacrylamide via Michael addition (Tian et al., 2012). Based on optimizing grafting ratio (Zhang et al., 2015), PEN (the number of grafted N-isopropylacrylamide to PEI-25K is 128.6) was selected and employed herein. The binding affinity of PEN to MT01 was examined using an agarose gel retardation assay. The migration of MT01 was completely retarded by PEN when the PEN/MT01 w/w ratio was over 0.5 (Figure 1A). Proper particle size and positively charged profile of PEN/MT01 nanoparticles were beneficial for endocytosis and transfection. The particle size and zeta potential of PEN/MT01 at different mass ratios were determined using a Malvern Nano ZS90 Zetasizer. With an increasing ratio of polymer to MT01, nanocomposites ranged from 146.33 ± 8.86 to 328.80 ± 9.06 nm in size, and the Zeta potential increased from −13.40 ± 0.74 mV at a ratio of 2.0 up to +15.93 ± 1.90 mV at 8.0 (Table 2). TEM images (Figure 1B) showed that PEN/MT01 nanocomposites had a globular morphology with a w/w ratio of 6.0. Figure 1C showed the particle size distribution of PEN/MT01 nanocomposites. The result showed that the particle size of PEN/MT01 nanocomposites had a normaldistribution curve with aunimodal distribution with a w/w ratio of 6.0.

**TABLE 2:** The particle size and zeta potential of PEN/MT01 complexes at different ratios.

PEN/MT01 ratios	Particle size (nm)	Zeta potential (mV)
2.0	328.80 ± 9.06	−13.40 ± 0.74
4.0	146.33 ± 8.86	+6.82 ± 0.33
6.0	151.07 ± 11.38	+12.67 ± 2.99
8.0	151.23 ± 3.02	+15.93 ± 1.90

Nanocomposite biomaterials, a relatively new class, incorporate biopolymeric and biodegradable matrix structures with bioactive and easily resorbable nanosized fillers (Pina et al., 2015). PEIs have been used in various applications in biomedicine, biotechnology, and biomaterial science. However, abundant cationic charges result in high transfection activity and loading capacity, and PEIs exert serious cytotoxic effects in living organisms (He et al., 2013). Specific advantageous features can be introduced through purposeful modification or functionalization (Mehdi et al., 2016). N-isopropylacrylamide modified the derivative PEN via Michael addition, and it had a higher transfection efficiency and lower cytotoxicity than PEIs. PEN-delivered siVEGF has a significant therapeutic effect on CT26 tumors (Chen et al., 2016). Moreover, Xing (Xing et al., 2020) employed PEN as a carrier for miR-29a transfection, which inhibited cell proliferation, migration, and invasion.

In this study, PEN was used as the carrier to deliver MT01. Effects of free MT01 and MT01-s on bone formation were tested as a control group. The ability to bind and condense DNA into nano-sized complex particles is a prerequisite for efficient gene delivery vectors. Our data suggested that neutral or positively charged complexes between PEN/MT01 were formed when the w/w ratio reached 0.5 (Figure 1). Intensity of MT01 band decreased with increasing mass ratio of PEN to MT01, indicating that PEN could condense MT01 into stable nanoparticles via electrostatic interaction. The hydrodynamic diameter and zeta potential of the PEN/MT01 nanocomposites were measured at various mass ratios (Table 2). In our previous study, we measured the images of nanocomposites (Zheng et al., 2017). When the w/w ratio reached 6.0, the hydrodynamic diameter of the PEN/MT01 nanocomposites was approximately 166 nm, and the zeta potential of the nanoparticles was positive. Taken together, these characteristics qualify the derivative PEN as a potentially efficient carrier for MT01 delivery.

### 3.2 PEN/MT01 nanocomposites had good biocompatibility in MC3T3-E1 cells

**FIGURE 2:**
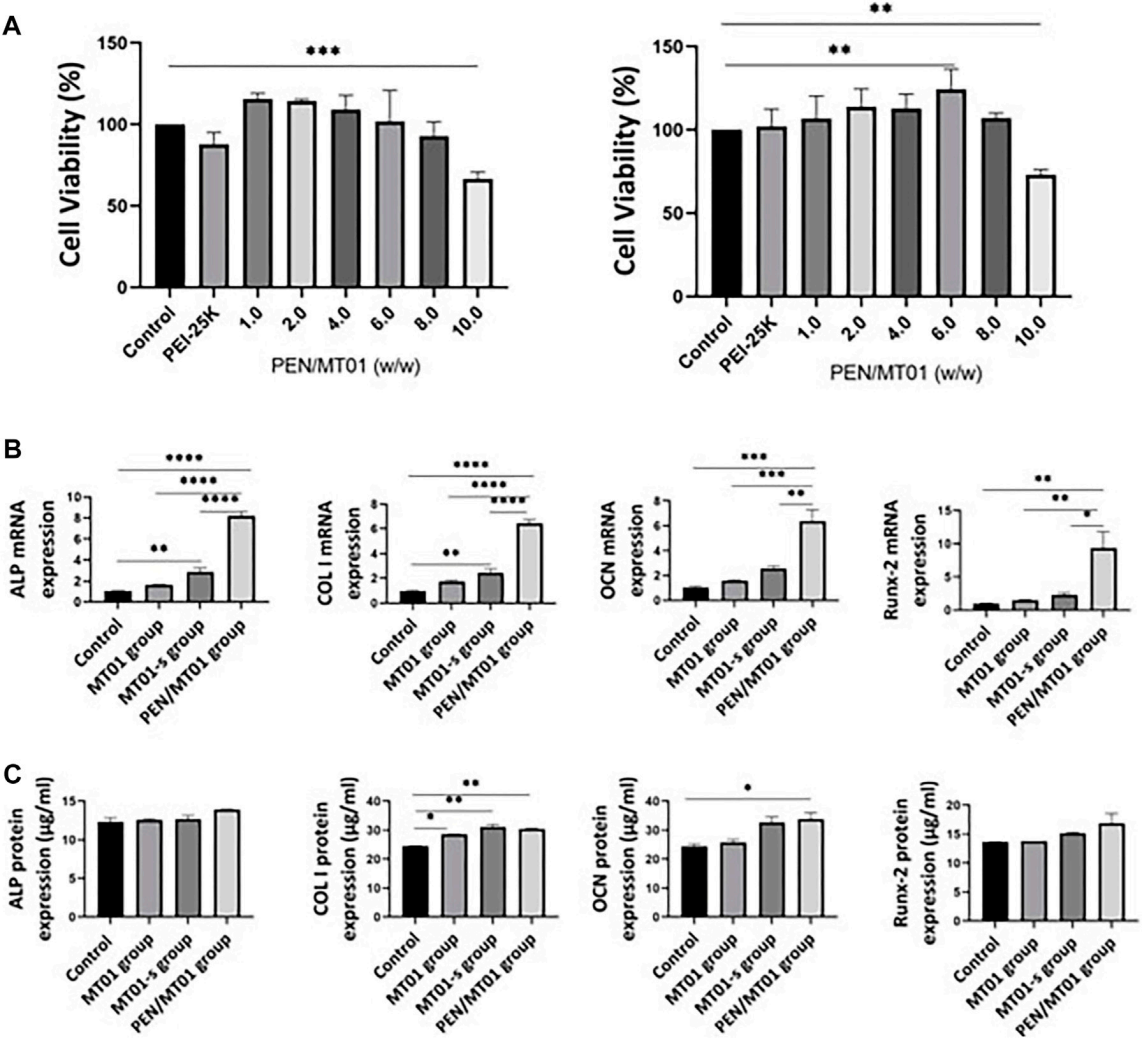
Biocompatibility and osteogenesis factor characteristics of PEN/MT01 nanocomposites. **(A)** Viability of MC3T3-E1 cells cultured with PEN/MT01 at different w/w ratios at 24 h and 48 h **(B)** ALP, COL I, OCN and Runx-2 mRNA expression in the control, MT01, MT01-s, PEN/MT01groups.The value is normalized by 2^^−ΔΔCt^ Method. **(C)** ALP, COL I, OCN and Runx-2 protein expression were measured by ELISA in the Control, MT01, MT01-s, PEN/MT01 groups. *: *p* < 0.05, **: *p* < 0.01, ***: *p* < 0.001, ****: *p* < 0.0001.

To determine the cytotoxicity of PEN/MT01 nanocomposites, MC3T3-E1 cells were transfected with PEN/MT01 complexed at different w/w ratios. In all groups, the control group was treated with PBS. When the mass ratio was lower than 8.0, the complex was non-toxic to cells (*p* > 0.05). When the ratio was 10.0, cytotoxicity was significant (*p* < 0.01, Figure 2A). When the mass ratio was 6.0, compared with other groups, the complex significantly increased MC3T3-E1 cell value (*p* < 0.01). The above results can provide guidance for the reasonable use of the maximum efficacy of PEN, so we finally selected a mass ratio of 6.0 for subsequent experiments.

According to the cell viability results (Figure 2A), PEN/MT01 nanocomposites is non-toxic, proving that nanocomposites can improve surface electricity of MT01, resist nuclease degradation *in vivo*, enhance transfection efficiency in cells, and achieve efficient delivery, which is consistent with our previous experimental results (Zheng et al., 2017).

### 3.3 PEN/MT01 nanocomposites promoted osteogenic differentiation markers expression

To further investigate how PEN/MT01 promotes the osteogenesis of MC3T3-E1 cells, the mRNA and protein expressions of osteogenesis-related genes were measured by q RT-PCR and ELISA after 7 days of differentiation. The control group was the same as the above treatment. MT01-s is a positive control group treated with chemical sulfur modification. q RT-PCR results demonstrated that free-MT01, MT01-s, and PEN/MT01 groups increased the mRNA expression levels of ALP, COL I, osteocalcin (OCN), and Runx-2 in MC3T3-E1 cells on the seventh day. However, the mRNA expression levels of ALP, COL I, OCN, and Runx2 in the free MT01 group and OCN and Runx-2 in the MT01-s group were not significantly different from those in the control group (*p* > 0.05). In contrast, mRNA expression levels of ALP, COL I in MT01-s group and ALP, COL I, OCN, and Runx-2 in PEN/MT01 group were significantly higher (*p* < 0.01, Figure 2B). Our enzyme-linked immunosorbent assay (ELISA) data demonstrated no difference in protein expression of ALP and Runx-2 between groups. However, COL I protein expression (*p* < 0.01) and OCN (*p* < 0.05) in PEN/MT01 group were significantly different from those in control group. Only COL I protein expression in MT01-s group was different from that in control group (*p* < 0.01, Figure 2C).

ALP, COL I, OCN, and Runx-2 are involved in bone tissue formation, metabolism, and regeneration and are often used as osteoblast differentiation markers (Stein et al., 2004; Yang et al., 2010; Rifai et al., 2017; Joanna et al., 2022). Herein, up-regulating these markers was closely associated with MT01-induced osteogenesis and implied that MT01 could directly improve the osteogenesis of cells.

Using chemically modified MT01-s or carrier-loaded PEN/MT01 can overcome the shortcomings of MT01 and play its role. This result is similar to our previous results (Hou et al., 2012). According to the comprehensive experimental results (Figure 2B), the osteogenic effect of PEN/MT01 was better than that of MT01-s. This proves that PEN can release MT01 slowly and prolong its action. ELISA results (Figure 2C) demonstrated an interesting phenomenon: the protein and mRNA expression differences differed in the same period, but the overall trend was similar. Generally, upregulation of gene expression occurs earlier than that of protein. This was because protein expression is related to mRNA levels and the regulation of translation and degradation. Herein, there was little difference in protein expression, which was more likely because it had not reached a peak in protein expression, and testing at the same date may lead to these differences because of this delay in actions. This may also mean that protein expression may not always be followed by the corresponding gene expression.

### 3.4 PEN/MT01 nanocomposites promoted osteoblastic differentiation

**FIGURE 3:**
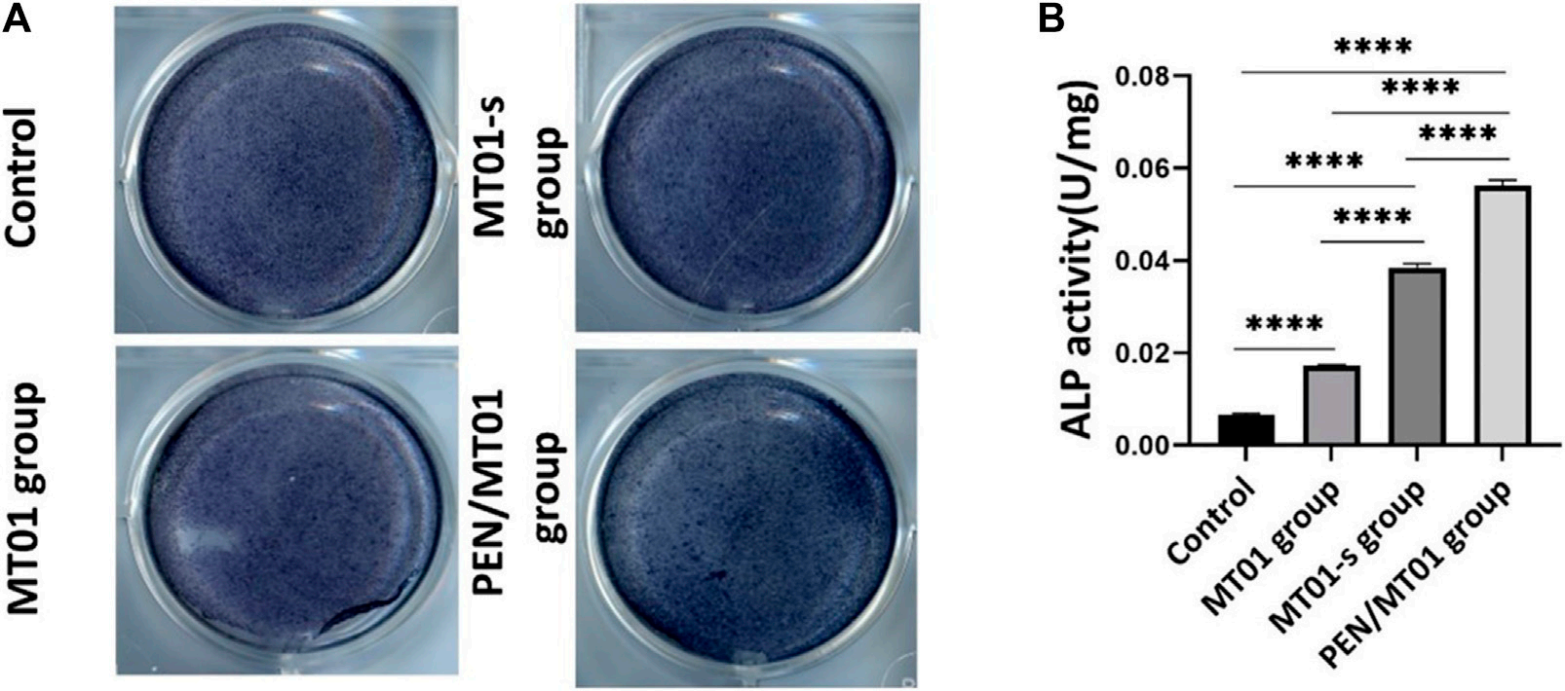
Effects of MC3T3-E1 cells osteogenic differentiation. **(A)** ALP Staining. **(B)** ALP quantitative assay. Data are presented as the mean ± SD, n = 3. ****: *p* < 0.0001.

The activity of osteogenic differentiation marker ALP is an important index to indicate osteogenesis at an early stage. The results demonstrated that PEN/MT01 nanoparticles significantly improved ALP activity compared to the control group (Figure 3A). ALP activity of MC3T3-E1 cells was measured after 7 days of treatment, and the results were illustrated (Figure 3B). Following the increased ALP mRNA expression in MC3T3-E1 cells, maintained in MT01-s and PEN/MT01, ALP activity increased after 7 days of treatment, whereas in free MT01, there was no statistically significant increase in ALP content following gene expression. Consequently, PEN/MT01 group increased mineralization and osteogenesis activity.

The positive ALP staining results indicated that PEN/MT01 complex had osteogenic potential. This improvement was related to control ability through efficient delivery of the carrier. Accordingly, results implied that MT01 could promote osteogenic differentiation of MC3T3-E1 cells, but osteogenic effect of free MT01 was non-significant or only had a certain effect in the initial stage, which is due to the unstable nature of MT01 itself and its easy degradation. Concurrently, MT01 has a negative charge and a low cell uptake rate.

### 3.5 PEN/MT01 nanocomposites had good effect on serum biochemical indexes *in vivo*

To detect the toxic effect of composite materials on rats *in vivo*, serum biochemical indicators were tested. Serum biochemical indices results displayed that, compared to control group, levels of GPT, GOT, and BUN of rats in each experimental group did not change significantly, indicating that PEN/MT01 did not affect the liver, kidneys, and other organs (*p* > 0.05, Table 3). Serum biochemical index results displayed that PEN/MT01 unaffected important organs function, including rats liver and kidney rats. It has the advantage of low toxicity and side effects; therefore, it can be used for bone repair and regeneration.

**TABLE 3:** Changes of GPT, GOT and BUN in rats of each group.

Group	GPT (IU/L)	GOT (IU/L)	BUN (mmol/L)
Control group	55.65 ± 6.56	203.76 ± 15.45	8.2 ± 1.3
MT01 group	56.41 ± 7.25	204.12 ± 13.12	7.97 ± 0.61
MT01-s group	55.61 ± 5.23	199.87 ± 18.59	7.6 ± 0.52
PEN/MT01 group	58.10 ± 8.32	201.32 ± 11.32	7.91 ± 0.84

### 3.6 Micro-CT depicted that PEN/MT01 nanocomposites promoted forming of new bone in defect

**FIGURE 4:**
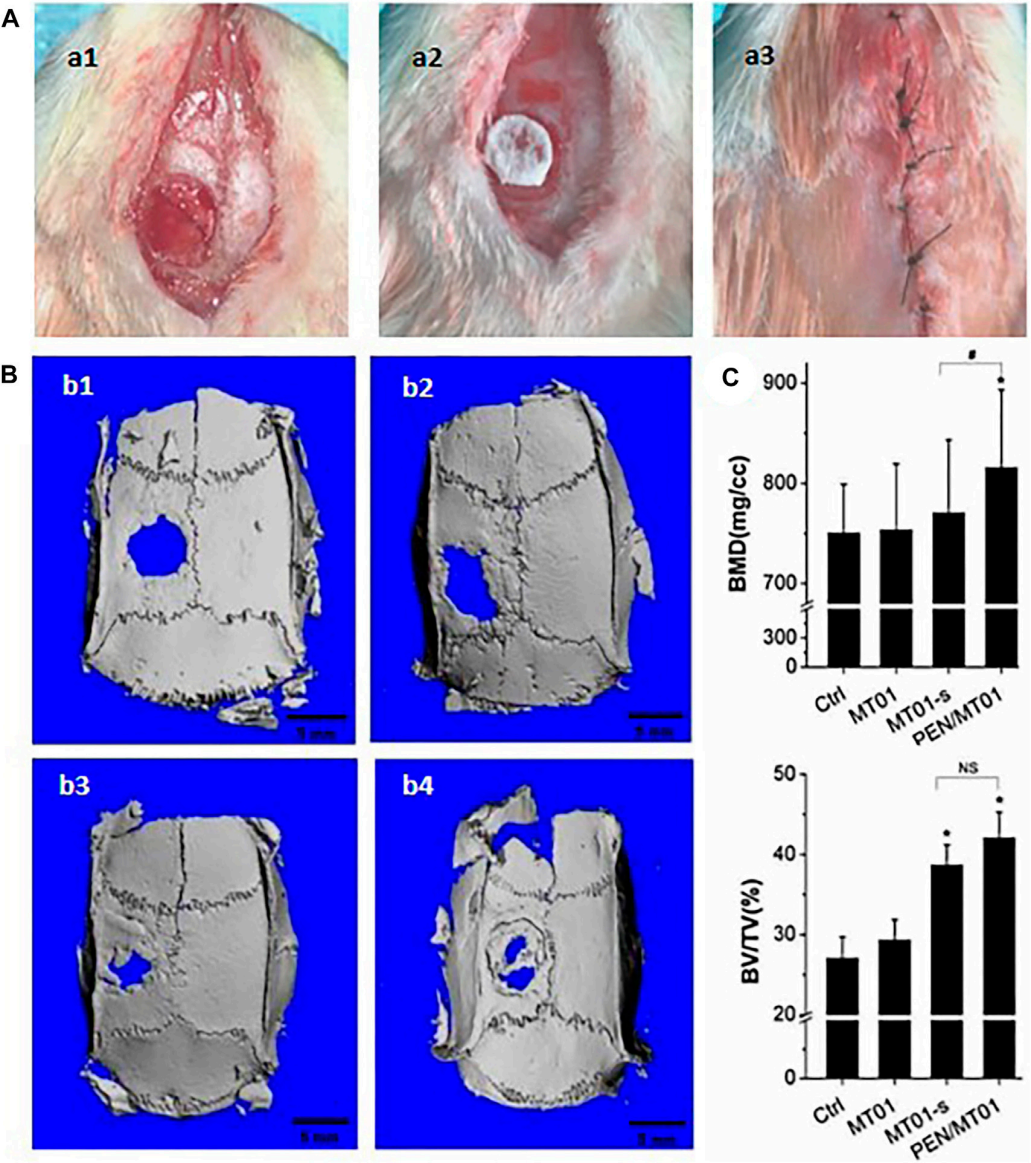
The rat skull defect model and osteogenesis effect of PEN/MT01 *in vivo*. **(A)** Operation of rat skull defect model. a1: skull defect in rats; a2: place the biofilm; a3: suture. **(B)** Micro CT 3D Reconstruction Image. b1: Control group; b2:MT01 group; b3:MT01-s group; b4: PEN/MT01 group, scale bar represents 5 mm. **(C)** Quantitative analysis of BMD and BV/TV determined by Micro-CT images. *: *p* < 0.05 (ststistical differences between other experimental groups and the control group), #: *p* < 0.05, NS: *p* > 0.05 (ststistical differences between the MT01-s group and PEN/MT01 group).

In this work the critical size defect technique was used to evaluate the systemic activity of free MT01, MT01-s, and PEN/MT01 on bone regeneration. In experimental studies, the calvarial defect model has been regarded as the most selective experimental model of bone regeneration because of the poor blood supply and the membranous structure precluding any spontaneous healing (Dupoirieux et al., 2001). In the present study, A 5 mm diameter hole was made in each parietal bone of male SD rats because the bony lesions above this critical size become scarred rather than regenerated, remaining of the cavity (Toker et al., 2012). This was confirmed by the lack of the bone regeneration at the control defects in our experiment. As displayed in Figure 4A, this is the rat skull defect model process. After killing rats at the end of period, micro-CT and histomorphometry were used to observe skull defects of rats in each group. Three-dimensional reconstruction image and volume measurement of micro-CT analysis showed that control and MT01 groups had slight osteogenesis at the edge of defect. In contrast, MT01-s and PEN/MT01 groups had obvious osteogenesis at the defect (Figure 4B). According to new bone mass analysis, BMD and BV/TV in MT01-s and PEN/MT01 groups were higher than those in blank control group. However, there was a non-significant difference between MT01 and blank control groups in BV/TV (Figure 4C, *p* < 0.05). BMD of compound group was the most obvious.

Rat skull defects are considered a rapid and effective method for evaluating bone tissue regeneration. MT01, MT01-s, and PEN/MT01 were loaded onto the oral biofilm and placed on the skull defect of rats to observe their osteogenic ability (Figure 4A). *In vivo* animal experiments demonstrated the beneficial *in vitro* properties of PEN/MT01, revealing accelerated healing and enhanced new bone formation within a cranial critical-sized defect in a rat model (Figures 4B, C). The analysis of newborn bone mass displayed that the BMD of the PEN/MT01 group was higher than that of the control and MT01 groups. Furthermore, observation of bone morphology of the defect area demonstrated that osteogenesis of the skull defect area of rats with MT01-s and PEN/MT01 was better than that of the MT01 and control groups.

### 3.7 H&E staining demonstrated that PEN/MT01 nanocomposites healed bone defect area well

**FIGURE 5:**
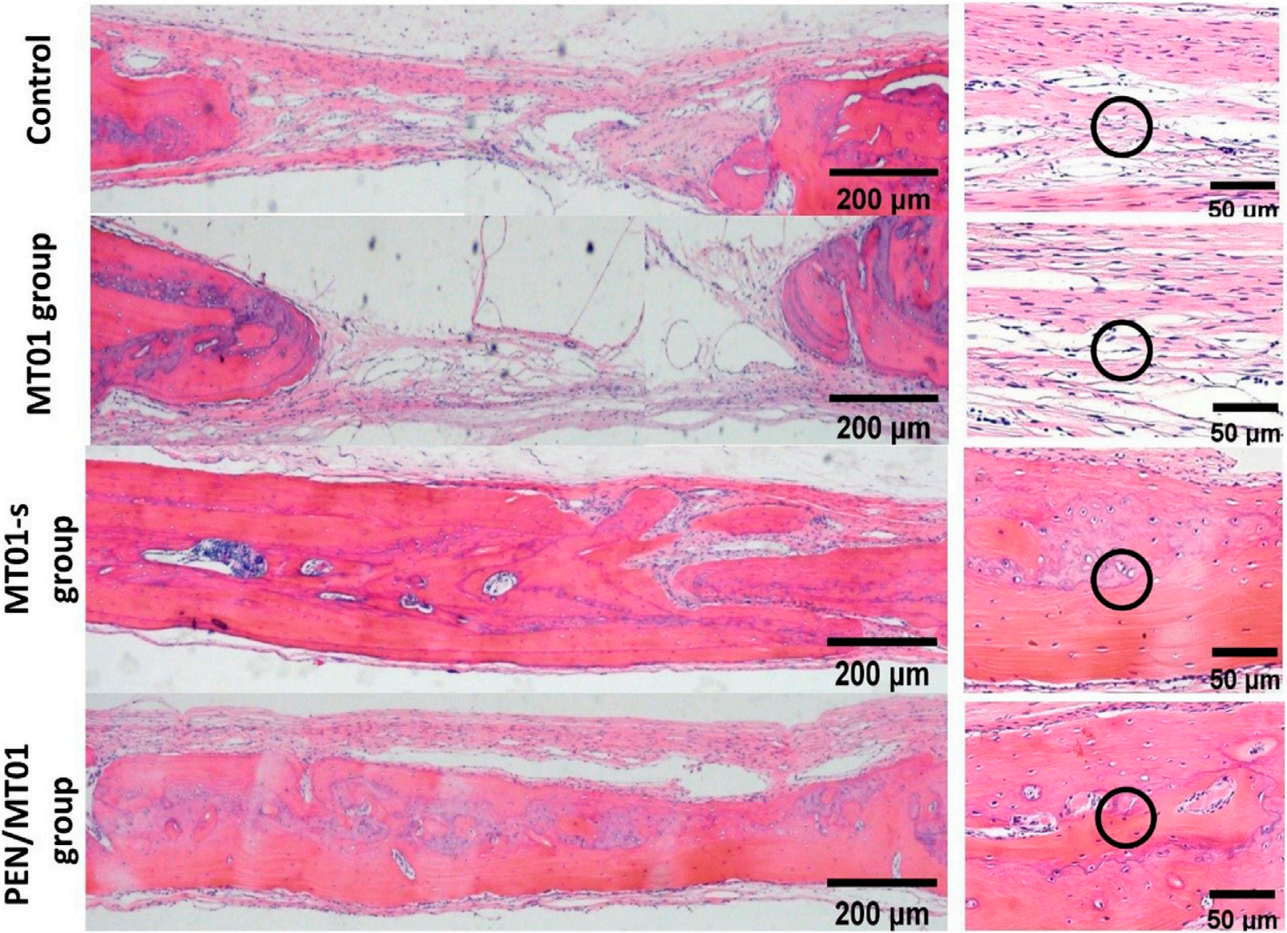
Histology analysis of the bone defect area in rats. H&E image of the bone defect area, scale bar represents 200 and 50 μm. The black circles indicate collagen fibers (the control and MT01 group) and newborn bone tissue (MT01-s and PEN/MT01 groups).

According to morphological observation results, after the materials of each experimental group were implanted into skull defect of rats, a small amount of new bone (mainly filled with fibrous connective tissue) was formed in control and MT01 groups 10 weeks after the operation, which remained in a defect state. New bone formation was observed in the defect area of MT01-s and PEN/MT01 groups, and host bone defect area healed well without obvious inflammatory reaction. PEN/MT01 group exhibited the best new bone formation (Figure 5).

H&E staining displayed that new bone formation was significantly enhanced in the PEN/MT01 group than in the other groups.

### 3.8 IHC staining showed that PEN/MT01 nanocomposites enhanced Runx-2 positive expression in cytoplasm

**FIGURE 6:**
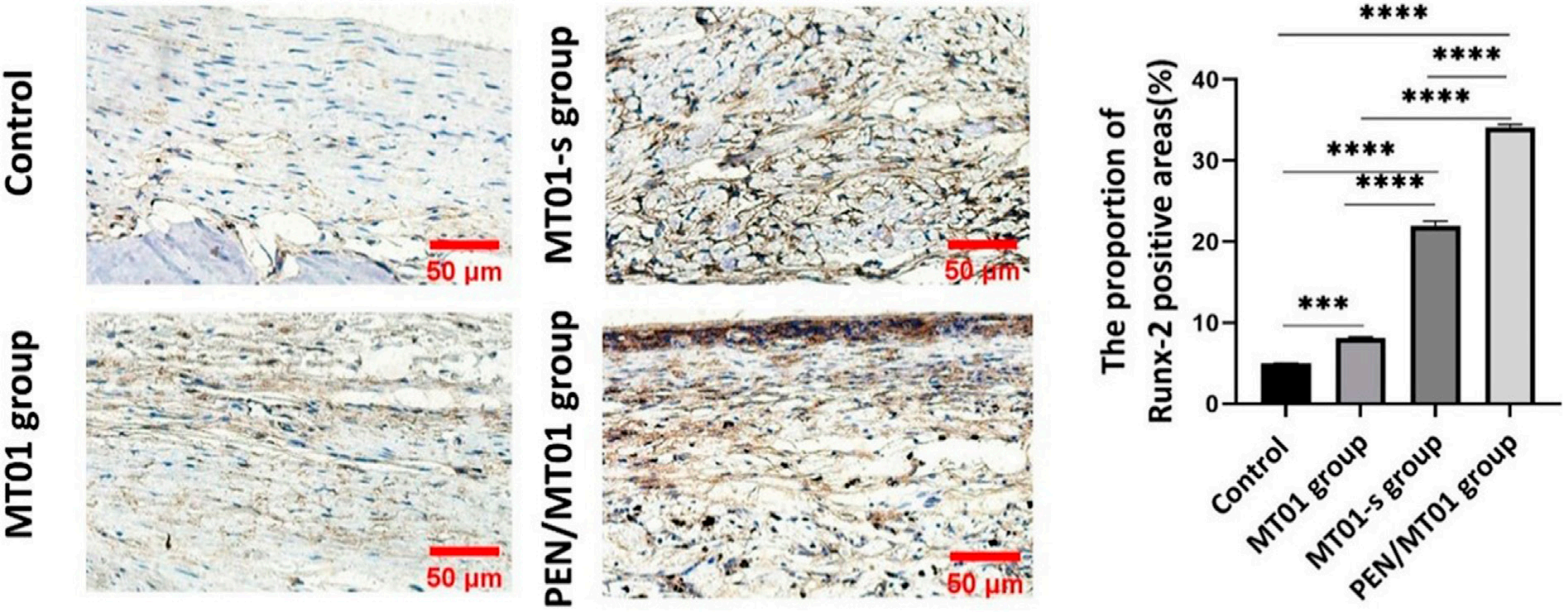
IHC analysis of the bone defect area in rats. **(A)** The results of IHC staining of Runx-2 in bone defect area of rats in each group after 10 weeks of implantation, scale bar represents 50 μm. **(B)** The positive areas of Runx-2 in stained bone defect area were analyzed using ImageJ software. Data are presented as the mean ± SD, n = 3 specimens/group, ***p < 0.001.

IHC staining of rat defect tissue sections with Runx-2 antibody (Figures 6A, B) showed that, under the same exposure rate, compared to the control group (5.00% ± 0.10%) and free MT01 group (8.14% ± 0.21%), there were obvious positive areas in the MT01-s and PEN/MT01 group, with proportions of Runx-2-positive areas up to 21.99% ± 1.00% and 34.12% ± 0.62%.And the results showed that Runx-2 expression in osteoblasts of rat skull tissue and cytoplasm near the periosteum in PEN/MT01 group was more significant than in other groups. IHC staining also revealed that Runx-2 expression in PEN/MT01 was higher than in the other groups (Figure 6). Therefore, PEN may be a good carrier of MT01 to promote osteoblast differentiation.

In summary, this study provides a new method to enhance the osteogenic influence by designing an effective delivery system, PEN, and delivering MT01. This is a new attempt to study the characteristics of these preparations in bone defects and to prove their effectiveness *in vivo*.

## 4 Conclusion

This study found that prepared PEN/MT01 has good biocompatibility and low toxicity. Following PEN-delivered MT01 transfection, osteoblastic differentiation of MC3T3-E1 cells and formation of new bone in the defect was efficiently promoted. Consequently, derivative PEN can potentially be used as an effective carrier for delivery of therapeutic oligonucleotides, which will greatly facilitate research and development of bone regeneration gene therapy.

## Data Availability

The original contributions presented in the study are included in the article/supplementary material, further inquiries can be directed to the corresponding authors.
